# *Cell Death Disease* turns 15: legacy of service, horizon of challenges

**DOI:** 10.1038/s41419-025-08325-1

**Published:** 2025-12-22

**Authors:** Gerry Melino, Yufang Shi, Hans Uwe Simon, Mauro Piacentini

**Affiliations:** 1 Department of Experimental Medicine, TOR, University of Rome “Tor Vergata”, Rome, Italy; 2 The Fourth Affiliated Hospital of Soochow University, Institutes for Translational Medicine, Suzhou Medical College, Soochow University, Suzhou, China; 3 Institute of Pharmacology, University of Bern, Bern, Switzerland; 4 Institute of Biochemistry, Brandenburg Medical School, Neuruppin, Germany; 5 National Institute for Infectious Diseases IRCCS “Lazzaro Spallanzani”, Rome, Italy

**Keywords:** Cell death, Pathogenesis

2025 marks the 15th anniversary of *Cell Death & Disease* (CDDis), a milestone that underscores the journal’s enduring impact and expanding influence in cell biology, pathology, and translational medicine. What promoted the founding of CDDis? What was the scientific context at the time? And why establish yet another journal focusing on cell death?

Between the late 80’s and early 90’s researchers witnessed the remarkable and incredible emergence of a novel scientific paradigm: programmed cell death. Building on Richard Lockshin’s early description of programmed cell death in insects [1], and John Kerr’s coining of the term “apoptosis” [2], scientists rapidly delineated a genetically- and biochemically-regulated form of cell death at the molecular level. This breakthrough sparked an explosion of discoveries, revealing its central role in both physiology and pathology [3]. The pivotal studies in the 80’s by H. Robert Horvitz on *C. elegans* elucidated the core molecular mechanisms [4]. These genetic pathways laid the groundwork for identifying analogues biochemical cascade in mammals: the enzyme CED-3 (CED, for cell death abnormal) [5], triggered a proteolytic cascade, precisely regulated by a specific protein, CED-4 and, in turn by CED-9. Human orthologues were swiftly identified thereafter.

In 1984, Carlo Croce’s group discovered BCL-2 [6]; whose anti-apoptotic function was elucidated by David Vaux in 1988 [7], and Stan Korsmeyer in 1989 [8], establishing BCL-2 as a powerful inhibitor of cell death and a functional homologue of CED-9 [9]. Caspases, the executioner proteases, were also identified and classified (reviewed in [10]). Amid this surge of insights into transformative biological process, the scientific community needed a dedicated forum for discourse. Thus *Cell Death and Differentiation* was launched, in 1994,quickly becoming the prominent venue for the field, a position it retained today [11–20].

Fifteen years later, as the molecular underpinning of cell death mature sufficiently for therapeutic application in disease from cancer to immunity and neurodegeneration, the journal’s editors recognized the need to broaden its scope toward translational research. With programmed cell death increasingly recognized as cornerstone of pathology across these domains, a specialized outlet was essential to bridge basic science and clinical relevance. In 2010, we launched its sister journal *Cell Death & Disease*. As a highlight this innovation, CDDis marked the first “sister journal” in the Nature Publishing Group (now Springer Nature) portfolio.

*Cell Death & Disease* was conceived as part of the CDD*press* journal family, CDDis has immediately established itself as a key platform for publishing high-quality research on the mechanisms and implications of cell death in human diseases. Over these 15 years, we believe *Cell Death & Disease* has succeeded in bridging the gap between basic cell biology and clinical research. It was envisioned as an open-access journal, an ambitious choice in the publishing landscape that proved prescient, anticipating the scientific community’s growing shift toward greater transparency and accessibility. This model has enabled us to reach a global audience of researchers, clinicians, and students, facilitating the dissemination of critical scientific findings across various biomedical disciplines. One piece of evidence of this “*success*” is the fact that two journals were not any more sufficient, that the field was ready to address therapeutic aspects, and that there was a need for yet another sister- journal. In fact, in 2015 we launched *Cell Death Discovery*. Over the past 15 years, CDDis has published more than 11,000 articles (circa 95% of which are original papers), that have substantially advanced our understanding of diverse cell death modalities (e.g. apoptosis, necroptosis, ferroptosis, entosis, disulfidoptosis, autophagy-related cell death) and their roles in disease processes. The journal’s high citation rates reflect its quality, relevance, and consistent scientific impact of the journal, as evidenced by its consistently high and stable impact factors, see Tables 1 and 2. The journal’s interdisciplinary scope has steadily expanded to include emerging fields such as inflammation, immunology, metabolism, and drug resistance. This development reflects the increasingly complexity and interconnectedness of contemporary biomedical research. With a steadily growing impact factor and a reputation for scientific rigor, *Cell Death & Disease* has established itself as a trusted n venue for publications by both seasoned and early-career researchers. Its editorial board, consisting of internationally renowned scientists, has upheld rigorous standard of peer review and editorial integrity.

**Table 1:** Most cited work published in *Cell Death & Disease*.

*first author*	*topics*	*Year*	*cites*	*Ref*
Li, J et al.	ferroptosis	2020	3186	[21]
Yang, Y et al.	inflammasome	2019	1124	[22]
Panieri E & Santoro MM	ROS	2016	1016	[23]
Volpe, CMO et al.	ROS	2018	1000	[24]
Gao, F et al.	mesenchymal stem cells	2016	975	[25]
Jiang, Y et al.	T-cells	2015	965	[26]
Fucikova, J et al.^a^	Immunogenic death	2020	805	[27]
Park, E & Chung, SW^a^	ROS ferroptosis	2019	775	[28]
Xia, XJ et al.	pyroptosis	2019	715	[29]
Liu, R et al.	PI3K/AKT	2020	685	[30]
**More Recent work**				
Liu, SZ et al.	autophagy	2023	510	[31]
Calabresi, P et al.	Parkinson’s	2023	350	[32]
Chen, Y et al.	ferroptosis	2023	300	[33]
Liu, YE et al.	Ferroptosis	2023	225	[34]
Zhao. Y et al.	tumor microenvironment	2023	205	[35]
An, XQ et al.	ROS	2024	175	[36]

^a^Original work.

**Table 2:** Impact Factor and ranking of *Cell Death & Disease*.

*Year*	*IF*	*Journal Ranking*
2024	9.6	29/204^*a*^
2023	8.1	33/205^*a*^
2022	9.0	31/191^*a*^
2021	9.7	36/195^*a*^
2020	8.5	37/195^*a*^

Journals’ Landscape		
*Journal*	*IF 2023*	*IF 2024*
Nature Cell Biology	17.3	19.1
Molecular Cell	14.5	16.6
Cancer Research	12.5	16.6
Nature Comms	14.7	15.7
***Cell Death Differentiation***	13.7	15.4
Science Advances	11.7	12.5
***Cell Death Disease***	8.1	9.6
PNAS-USA	9.4	9.1
EMBO Journal	9.5	8.3
Oncogene	6.9	7.3
***Cell Death Discovery***	6.1	7.0
Cell Reports	7.5	6.9
Sci Signal	6.8	6.6
Biology Direct	5.7	4.9
Molecular Oncology	5.0	4.5
FEBS Journal	5.5	4.2

^a^data from Clarivate.

data from Clarivate.

As *Cell Death & Disease* celebrates 15 years of editorial excellence, its future looks bright. We will engage the community in discussions of challenges head, such as the evolving ethics of publishing and mounting pressure to quantify scientific career. Artificial Intelligence is already affecting changes across the scientific landscape at multiple levels, warranting proper discussion. Last but not least, emerging new geographic powerhouse, such as China, with India soon to follow, will strongly reshape the global research ecosystem. We are ill-prepared for these shifts, making robust community engagement essential. The journal continues to innovate by exploring new publication formats, promoting data transparency, and advancing diversity in science. CDDis remains committed to supporting the next generation of researchers, fostering interdisciplinary dialogue, and advancing our understanding of how cell death contributes to disease and therapy. In an era of rapid biomedical advancement, *Cell Death & Disease* stands as a high-quality forum for scientific discovery, discourse, collaboration, and impact.
